# Development and psychometric testing of a barriers to HIV testing scale among individuals with HIV infection in Sweden; The Barriers to HIV testing scale-Karolinska version

**DOI:** 10.1186/s12955-015-0381-7

**Published:** 2015-11-19

**Authors:** Maria Wiklander, Johanna Brännström, Veronica Svedhem, Lars E. Eriksson

**Affiliations:** Department of Neurobiology, Care Sciences and Society, Karolinska Institutet, Stockholm, Sweden; Department of Clinical Sciences Danderyd Hospital, Karolinska Institutet, Stockholm, Sweden; Department of Infectious Diseases, Karolinska University Hospital, Stockholm, Sweden; Department of Medicine Huddinge, Karolinska Institutet, Stockholm, Sweden; Department of Learning, Informatics, Management and Ethics, Karolinska Institutet, Stockholm, Sweden; School of Health Sciences, City University London, London, UK; Division of Nursing, Department of Neurobiology, Care Sciences and Society, Karolinska Institutet, 23300, SE-141 83 Huddinge, Sweden

**Keywords:** HIV, HIV testing, Health Services Accessibility, Prevention, Attitudes, Questionnaires, Psychometrics

## Abstract

**Background:**

Barriers to HIV testing experienced by individuals at risk for HIV can result in treatment delay and further transmission of the disease. Instruments to systematically measure barriers are scarce, but could contribute to improved strategies for HIV testing. Aims of this study were to develop and test a barriers to HIV testing scale in a Swedish context.

**Methods:**

An 18-item scale was developed, based on an existing scale with addition of six new items related to fear of the disease or negative consequences of being diagnosed as HIV-infected. Items were phrased as statements about potential barriers with a three-point response format representing not important, somewhat important, and very important. The scale was evaluated regarding missing values, floor and ceiling effects, exploratory factor analysis, and internal consistencies.

**Results:**

The questionnaire was completed by 292 adults recently diagnosed with HIV infection, of whom 7 were excluded (≥9 items missing) and 285 were included (≥12 items completed) in the analyses. The participants were 18–70 years old (mean 40.5, SD 11.5), 39 % were females and 77 % born outside Sweden. Routes of transmission were heterosexual transmission 63 %, male to male sex 20 %, intravenous drug use 5 %, blood product/transfusion 2 %, and unknown 9 %. All scale items had <3 % missing values. The data was feasible for factor analysis (KMO = 0.92) and a four-factor solution was chosen, based on level of explained common variance (58.64 %) and interpretability of factor structure. The factors were interpreted as; *personal consequences*, *structural barriers*, *social and economic security*, and *confidentiality*. Ratings on the minimum level (suggested barrier not important) were common, resulting in substantial floor effects on the scales. The scales were internally consistent (Cronbach’s α 0.78–0.91).

**Conclusions:**

This study gives preliminary evidence of the scale being feasible, reliable and valid to identify different types of barriers to HIV testing.

## Background

About 35 million individuals in the world are currently living with HIV infection [1]. Of these, an increasing number have access to antiretroviral treatment (ART), which has substantially improved survival where treatment is available [2–4]. Early detection of HIV infection is vital for both treatment and prevention. Timely initiation of ART increases survival [2, 5] and significantly reduces the risk of further transmission [6]. In addition, people who are aware of their HIV infection often make behavioral changes to reduce the risk of onward transmission of HIV [7].

In Sweden, ART is generally accessible at no cost for all who are diagnosed with HIV and eligible for treatment. Despite this, a majority are diagnosed late [8], i.e. after when treatment is recommended to start according to national guidelines [9]. Identification of potential barriers to HIV testing is important for development of relevant strategies to promote testing and reach individuals with undiagnosed HIV infection. Health care professionals could benefit from knowing what patients perceive as barriers to HIV testing, as this knowledge could direct them on how to more actively initiate and encourage testing among patients. On the societal level, knowledge about existing barriers can give guidance on relevant targets for HIV prevention on a structural level (e.g. laws, infrastructure).

Well-known barriers to HIV testing include perceived low risk of HIV infection, structural barriers, concerns about confidentiality, and fears of the disease or of negative consequences of being diagnosed as HIV-infected, such as HIV-related stigma (for reviews of the literature, see [10–13]). It is advantageous to use structured and psychometrically tested instruments to assess barriers to HIV testing, since such instruments are evaluated for their qualities and give comparable results about the existence and magnitude of different barriers in various contexts. A few specific instruments to measures barriers to HIV testing have been published [14–17] of which only the scale by Awad et al. [14] has been evaluated for psychometric properties. There is also one psychometrically tested scale on attitudes to HIV testing which investigates both barriers and positive attitudes to HIV testing [18, 19]. The existing scales constitute an important basis for further investigation of barriers to HIV testing. However, our clinical experience from HIV health care in Sweden suggests that the existing scales do not include all barriers that are relevant, why an extended scale is needed. Therefore, the aims of the present study were to develop and test a barriers to HIV testing scale relevant for a Swedish context.

## Methods

### Design

This was a cross-sectional study within the Swedish national project “Late Presentation of HIV-1 infection” lead by Karolinska University Hospital, Sweden. Aim of the national project was to identify factors in HIV-infected patients and the health care system that contribute to late diagnosis. The project is described elsewhere [20]. Eligible for participation were adults living in Sweden, diagnosed with HIV infection from October 2009 to January 2012, with data collected within six months after their diagnosis.

### Instrument development

For the present study, an 18-item scale was developed, based on twelve items from the existing barriers to HIV testing scale by Awad et al. [14] and six new items. Dimensions measured in the original scale are *structural barriers*, *fatalism/confidentiality concerns*, and *fears*. The scale was evaluated and translated into Swedish by a bilingual panel of HIV experts. One item concerning costs of treatment was excluded in the new scale due to the general availability of treatment for free in Sweden. The six new items were added, based on the literature and clinical experiences, to expand on different feared consequences of being diagnosed with HIV [12, 13, 20]. Appropriateness of the content and phrasings of the new items was discussed with professionals from participating clinics. Three of the new items concerned potential consequences in social contacts and relationships of being diagnosed with HIV: fear of losing one’s family or friends (e.g. [21–24]) and fear of negative consequences in sexual life [25]. The remaining three items concerned other potential negative consequences for the individual of being diagnosed with HIV infection: fear of becoming ill [12, 13], worries about legal consequences [10] and fear of feeling like a failure. The topic of fear of legal consequences has been shown relevant from an international perspective [10] and is, according to clinical experiences and previous research [26] also relevant for a Swedish context where people living with HIV under the law are obligated to disclose their HIV status to sexual partners and when seeking health care. The eighteen items were phrased as statements about potential barriers and the respondents are instructed to rate the importance of the barriers described on a three-point scale from 0–2. The response alternatives and their respective scores are *not important* (0), *somewhat important* (1), and *very important* (2). The simple response format was chosen to make the scale feasible for a broad population of respondents, including people with limited language skills and literacy. Swedish and English versions of the scale were developed simultaneously (new items in English adapted by the bilingual expert panel). Items in the scale, titled the Barriers to HIV Testing Scale – Karolinska version, are presented in Table 1.

**Table 1:** Items, and factor loadings based on principal axis factoring with oblimin rotation (pattern matrix) in the Barriers to HIV Testing Scale – Karolinska version (*N* = 258)

Item	Factors			
	**1** ^a^	**2** ^b^	**3** ^c^	**4** ^d^
16. I was afraid of becoming sick	**.823**	.122	.103	-.077
15. I was afraid that my sex life would be negatively affected	**.809**	-.044	-.131	.121
18. I was worried about feeling like a failure	**.691**	.078	.048	-.230
14. I was afraid of losing my friends and other social contacts	**.480**	-.116	-.358	-.224
12. I was afraid of losing my partner	**.455**	.029	-.395	-.057
17. I was worried about the legal consequences	**.450**	.068	-.341	-.085
9. I did not want to know the results	**.399**	.326	.033	-.222
4. The testing site was too far away	.016	**.699**	.033	.060
1. I did not have transportation to a testing site	.029	**.663**	.051	-.046
8. There was no cure so why get tested	-.025	**.634**	.036	-.266
3. I did not have enough time	.078	**.632**	-.081	.157
2. I did not know where to go for testing	-.118	**.532**	-.303	-.039
5. I did not like people at testing site	.085	**.344**	-.092	-.169
11. I was afraid of losing my job	-.011	.050	**-.795**	-.081
13. I was afraid of losing my family	.206	.097	**-.582**	-.146
10. I was worried about my insurance/insurances	.123	.147	**-.488**	-.092
6. I was worried about confidentiality	.030	.077	-.185	**-.744**
7. People might recognize me at testing site	.230	-.046	-.141	**-.550**

The factors were interpreted as relating to: ^a^ *personal consequences*, ^b^ *structural barriers*, ^c^ *social and economic barriers*, ^d^ *confidentiality*

Items assigned to scale with highest loading (in bold)

### Participants

Patients from three of the largest HIV clinics in Sweden and eight county clinics distributed throughout the country were eligible for participation in the present substudy. Inclusion criteria were being ≥ 18 years old and diagnosed with HIV infection up to 6 month before completion of the questionnaire. Since a majority of those living with HIV infection in Sweden are migrants and since individuals not speaking and understanding a main language are often excluded from research, an explicit goal of the study was to also include individuals with low comprehension of Swedish.

**Fig. 1:**
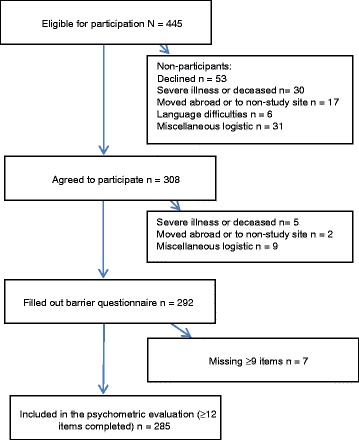
Flowchart of inclusion of participants in the study

The inclusion process is presented in Fig. 1. Of 445 eligible participants, 308 chose to participate in the study, and 292 completed the barrier questionnaire. Seven participants had missing values on half of the items or more and were excluded. The remaining 285 individuals had completed at least two thirds of the items and were included in the psychometric evaluation. The participants were between 18 and 70 years old (mean 40.5, SD 11.5), 39 % were females and 77 % born outside Sweden. Routes of transmission were heterosexual transmission (HT) 63 %, male to male sex (MSM) 20 %, intravenous drug use (IDU) 5 %, blood product/transfusion 2 %, and unknown 9 % (Table 2). Sixteen percent of the participants completed the Swedish version of the questionnaire with assistance of a professional interpreter (in-person or by telephone), who translated the items into the respondents’ language of origin. The English version of the questionnaire was used by 27 % of the participants. Participants did not differ from non-participants regarding sex, age or route of transmission, but were less likely to be born outside Sweden (OR 0.51, CI 0.32–0.80, *p* 0.004). Furthermore, the participants were representative for the total population of individuals with newly diagnosed HIV in Sweden during the study period (*N* = 827) [27] with regard to gender and origin, but were slightly older (mean age 40.5 vs. 38.9, *t*(284) 2.35, *p* 0.020) and had a lower representation of individuals with MSM as route of transmission (OR 0.68, CI 0.49–0.94, *p* 0.018).

**Table 2:** Description of the participants: percentages of sex, origin and paths of transmission (*N* = 285)

	%
Women	39
Origin	
Sweden	23
Other countries	77
Path of transmission	
Heterosexual	63
Male to male sex	20
Intravenous drug use	5
Blood product/ transfusion	2
Unknown	9

### Procedures

Eligible patients were informed about the study by their treating physician in connection with a visit at their outpatient clinic. They were given oral and written information about the purpose of the study and the voluntary nature of participation. The barrier questionnaires were administered by staff at the clinic, who could also answer questions regarding the questionnaire. Professional interpreters were available in-person or by telephone for participants who did not speak Swedish or English. Demographic data was collected from the Swedish InfCare HIV registry [27]. The study was approved by the Regional Ethical Review Board of Stockholm, Sweden (2009/1029-31/1-4).

### Statistical analyses

Participants were compared with non-participants and population data with Pearson’s chi-square tests for dichotomous data (each route of transmission dichotomized as present/absent) and t-tests for continuous data (independent two-samples *t*-test and one-sample *t*-test for comparisons with non-participants and population data, respectively). Data quality was assessed by inspection of missing values, means and standard deviations as well as floor and ceiling effects. Items were considered feasible if they had less than 3 % missing values [28]. Questionnaires with at least two thirds of the items completed were considered acceptable for the data analyses, except for the factor analysis which was calculated on complete questionnaires. Evidence for construct validity was investigated with exploratory factor analysis. The sample size was considered adequate for exploratory factor analysis of the 18-item questionnaire [29]. The Kaiser-Meyer-Olkin measure of sampling adequacy (KMO) [30] and Bartlett’s test of sphericity were used to determine adequacy of the data for factor analysis [31]. Factors were extracted with principal axis factoring. Oblimin rotation was used since the factors were expected to be related [32]. The final factor solution was based on comprehensibility and interpretability together with level of explained variance. Scales were constructed from the factors, where items with loadings ≥ 0.32 [32] were assigned to the scale with the highest loading. Items with loadings ≥ 0.32 on two or more scales were considered as cross-loading items [32]. Scale means were calculated by averaging completed items on each scale. Scale reliabilities were assessed with Cronbach’s α [33], where the reliability was interpreted as; α ≥ 0.9 excellent, 0.7 ≤ α < 0.9 good, 0.6 ≤ α < 0.7 acceptable, 0.5 ≤ α < 0.6 poor, and α < 0.5 unacceptable [34]. Bivariate correlations between scales were calculated with Spearman’s rho. All statistical analyses were conducted with IBM SPSS 22 (IBM Corp., Armonk, NY).

## Results

### Feasibility

All items were well accepted by the responding patients with less than 3 % missing values for each item.

### Factor analysis

The exploratory factor analysis (Table 1) was based on 258 complete questionnaires. The data was judged feasible for factor analysis (KMO 0.916, Bartlett’s test of sphericity χ[153] 2768.91, *p* <0.001). The factor analysis with principal axis factoring and oblimin rotation enabled different factor solutions. Eigenvalues > 1 suggested a three factor solution and solutions with three to five factors were evaluated. A four-factor solution, explaining 58.6 % of the common variance, was finally chosen based on interpretability and level of variance explained. Four items had cross-loadings (loadings > 0.32). Fear of losing one’s partner, fear of losing one’s friends and other social contacts, and worries about legal consequences, loaded most strongly on the *personal consequences* factor but also had substantial loadings on the *social and economic security* factor. Not wanting to know the results loaded most strongly on *personal consequences* but also had substantial loading on *structural barriers*. The assignment of cross-loading items to the factor with its highest loading was further motivated by the content of these items, which was judged to correspond well with the factor it was assigned to. The suggested factors are presented below (new items marked with *).

#### Factor 1, Personal consequences

This factor consisted of seven potential barriers relating to fear of consequences for the individual; fear of becoming ill*, fear of negative consequences for sexual life*, worries about feeling like a failure*, fear of losing friends and social contacts*, fear of losing partner, worries about legal consequences*, and not wanting to know the result.

#### Factor 2, Structural barriers

This factor consisted of six potential barriers relating to external barriers; not having transportation to a testing site, not knowing where to go for testing, not having enough time, too long distance to the testing site, not liking people at the testing site, and not testing because there is no cure.

#### Factor 3, Social and economic security

This factor consisted of three potential barriers concerning fear of losses related to job, family*, and insurances. Common for these barriers was that they are related to the social and economic security for both the individual and his or her family.

#### Factor 4, Confidentiality

This factor consisted of two potential barriers concerning confidentiality; worries about confidentiality and fear of being recognized at the testing site.

### Scale characteristics

Means, standard deviations, and floor and ceiling effects for the four scales are presented in Table 3. All response alternatives were used for all items, but the response alternative “not important” was most frequently used, resulting in substantial floor effects on all scales. Approximately one third of all respondents reported that none of the barriers had importance for their decision to get HIV tested. The bivariate correlations between the scales are presented in Table 4. Moderate to strong positive correlations (Spearman’s rho 0.478–0.709) were found between the subscales.

**Table 3:** Descriptive statistics for the Barriers to HIV Testing Scale – Karolinska version: number of items per scale, means, standard deviations (SD), floor and ceiling effects, and Cronbach’s α (*N* = 285)

Scale^a^	No. of items	Mean	SD	Floor/ceiling effects (%)^b^	α
Personal consequences	7	0.47	0.61	48.8/3.5	0.91
Structural barriers	6	0.24	0.40	58.2/0.7	0.78
Social and economic security	3	0.31	0.57	68.8/5.3	0.80
Confidentiality	2	0.48	0.70	61.4/11.9	0.80

^a^Possible range for all scales: 0–2, higher levels indicating more barriers

^b^Percentage of ratings at the lowest/highest possible score

**Table 4:** Bivariate Spearman’s rank correlations between the scales in the Barriers to HIV Testing Scale –Karolinska version (*N* = 285)

Scale	1	2	3
1. Personal consequences	-		
2. Structural barriers	0.524***	-	
3. Social and economic security	0.709***	0.518***	-
4. Confidentiality	0.708***	0.478***	0.568***

***p <0.001

### Reliability

The internal consistencies of the scales were acceptable to excellent (*personal consequences* α 0.91, *structural barriers* α 0.78, *social and economic barriers* α 0.81, and *confidentiality* α 0.81).

## Discussion

The present study shows preliminary feasibility, reliability, and internal validity of the 18-item Barriers to HIV Testing Scale – Karolinska version, measuring four dimensions of barriers to HIV testing at the individual level: *personal consequences*, *structural barriers, social and economic security*, and *confidentiality concerns.* Structural barriers and confidentiality concerns are well known barriers to HIV testing and these factors were similar to those in the original 13-item barrier scale by Awad et al. [14]. Fear is also a well-known barrier to HIV testing, including fear of social and economic losses, fear of being stigmatized and fear of becoming ill. In the Barriers to HIV Testing Scale – Karolinska version, fear and worries were reflected in two subscales, *personal consequences* and *social and economic security*. The two subscales are interpreted as mirroring two facets of fear for negative consequences of being diagnosed with HIV. The *personal consequences* scale is interpreted as reflecting fear relating to identity and personal needs, and the *social and economic security* scale is interpreted as reflecting fear on a more tangible level, relating to the social and economic security for the individual and his or her family. Since the two scales were highly correlated, it remains to test the scales’ predictive value in future studies to prove the usefulness of retaining two separate fear scales [28].

Substantial floor effects, reflecting a high proportion of participants reporting no barriers on the dimension measured, were found on all scales. A high rate of responses on the minimum level is problematic for instruments intended to evaluate small differences between groups or individuals, or changes on individual levels over time [28]. However, the Barriers to HIV Testing Scale – Karolinska version was developed mainly to identify types of barriers in different populations and different contexts. Low ratings on a scale in a population would just indicate that the type of barrier measured is not a major obstacle to HIV testing in that population. On the other hand, low ratings on all suggested barriers, as in the present sample, might also be a reflection of low perceived risk. Low perceived risk has been identified as a major barrier to HIV testing among groups with increased HIV prevalence [10, 12, 13]. It is difficult to compare our results with other studies, since the number of negative responses are seldom summarized and reported. However, in a study of barriers to HIV testing among individuals concurrently diagnosed with HIV and AIDS by Mills et al. [16] a majority of the respondents endorsed only “not perceiving oneself to be at risk for HIV” from a list of suggested barriers. Similarly, in the study by Awad et al. [14] the highest mean scale score was 1.54 on a scale from 1 (not important) to 3 (very important), implying that it was common on all the three scales, to experience a suggested barrier as not important for the decision not to test for HIV. Low perceived risk was not the focus of the present study, but from a HIV prevention perspective, mapping and quantification of people’s perception of risk appears important to investigate together with the measurement of other barriers. Future studies might consider the inclusion of items covering low perceived risk to develop the scale further.

Furthermore, the Barriers to HIV Testing Scale – Karolinska version was adapted for individuals living in Sweden and an item relating to treatment cost from the scale by Awad et al. [14] was excluded since HIV treatment is free of cost in Sweden. Although the item lacks relevance in Sweden, future studies might consider the inclusion or exclusion of a treatment cost item based on its relevance in the context studied.

This study aimed at including a representative sample of newly diagnosed individuals with HIV in Sweden. Therefore, individuals with limited knowledge in Swedish were purposely included. This strategy results in possible limitations as well as strengths of the study. The additional use of English versions and interpreters for those who did not speak Swedish could be considered a methodological weakness. However, the items were short straightforward statements and the expert panel had no difficulty in coming to agreement on the proper translation of items into Swedish or English. A definite strength was that all groups living with HIV in Sweden today were well represented. Of those who agreed to participate in the study, a large percentage completed the questionnaire with relatively few missing items, which indicates that the scale is comprehensible and acceptable for the relevant populations.

## Conclusions

The 18-item Barriers to HIV Testing Scale – Karolinska version shows adequate psychometric properties to identify different types of barriers to HIV testing. This study adds to previous research by offering an instrument that distinguishes between feared personal consequences for the individual and feared social and economic consequences that might involve both the individual and her or his family.
